# Clearance dynamics of lactate dehydrogenase and aldolase following antimalarial treatment for *Plasmodium falciparum* infection

**DOI:** 10.1186/s13071-019-3549-x

**Published:** 2019-06-10

**Authors:** Mateusz M. Plucinski, Peter D. McElroy, Pedro Rafael Dimbu, Filomeno Fortes, Doug Nace, Eric S. Halsey, Eric Rogier

**Affiliations:** 1 Malaria Branch, Centers for Disease Control and Prevention, Atlanta, Georgia USA; 2 U.S. President’s Malaria Initiative, CDC, Atlanta, Georgia USA; 3 National Malaria Control Program, Ministry of Health, Luanda, Angola

**Keywords:** Malaria, Antigen, Rapid diagnostic test, HRP2

## Abstract

**Background:**

Lingering post-treatment parasite antigen in blood complicates malaria diagnosis through antigen detection. Characterization of antigen clearance dynamics is important for interpretation of positive antigen detection tests.

**Results:**

We used a bead-based serological assay to measure lactate dehydrogenase (LDH), aldolase (Aldo), and histidine-rich protein 2 (HRP2) levels in 196 children with *Plasmodium falciparum* malaria treated with effective antimalarials and followed for 28 to 42 days as part of therapeutic efficacy studies in Angola. Compared to pre-treatment levels, antigen concentrations two days after treatment declined by 99.7% for LDH, 96.3% for Aldo, and 54.6% for HRP2. After Day 2, assuming a first-order kinetics clearance model, half-lives of the antigens were 1.8 days (95% CI: 1.5–2.3) for LDH, 3.2 days (95% CI: 3.0–3.4) for Aldo, and 4.8 days (95% CI: 4.7–4.9) for HRP2.

**Conclusions:**

LDH and Aldo show substantially different clearance rates than HRP2, and their presence is largely indicative of active infection.

**Electronic supplementary material:**

The online version of this article (10.1186/s13071-019-3549-x) contains supplementary material, which is available to authorized users.

## Background

Malaria diagnosis worldwide often relies on detection of parasite antigen using rapid diagnostic tests (RDTs) [1]. Most commonly, the target is the *Plasmodium falciparum*-specific histidine-rich protein 2 (HRP2), but other targets include lactate dehydrogenase (LDH) and aldolase (Aldo) [1, 2]. However, unlike a case confirmed through malaria microscopy, a positive RDT result does not necessarily imply an active infection because it may merely represent lingering antigenemia after successful clearance of parasites from the host.

As a consequence, the presence of parasite antigens in a person’s blood (leading to a possible RDT positive result) must be interpreted in the context of the clearance dynamics of these antigens. These dynamics have been well-described for HRP2, whose clearance obeys a first order kinetics model, with a half-life in the human host in the 3–5 day range [3, 4]. As a result, individuals can be HRP2-positive for weeks following adequate clearance of parasitemia [5, 6]. HRP2 clearance dynamics are highly consistent among different groups and parasite genotypes; deviations may indicate inadequate clearance of parasitemia in drug trials [3, 7]. Previous studies investigating the persistence of other *P. falciparum* antigens have found positive LDH-based RDT results for up to two weeks following successful parasite clearance [8, 9], though no LDH positive blood samples were identified 7 days post-treatment in an ELISA-based study [10]. To date, the longitudinal dynamics of *Plasmodium* Aldo in the human host has not been investigated. Information about clearance kinetics for these targets could inform interpretation of laboratory test results, particularly in persons who have recently received treatment. Here, we use a recently developed bead-based assay for laboratory antigen detection and quantification to characterize clearance dynamics of LDH and Aldo in individuals treated for *Plasmodium falciparum* infection.

## Methods

### Sample collection

We analyzed samples previously collected as part of therapeutic efficacy studies in Angola in 2015 [11] and 2017 [12]. Children with uncomplicated *P. falciparum* infection were treated with one of three artemisinin-based combination therapies: artemether–lumefantrine (AL), artesunate–amodiaquine (ASAQ), or dihydroartemisinin–piperaquine (DP), and followed for 28 days (AL and ASAQ) or 42 days (DP). Blood was collected on Whatman 903 filter paper (GE Healthcare, Chicago, IL, USA) on Day 0, Day 2 (2017 only), Day 3, Day 7, and weekly thereafter. Three-day treatment was given starting on Day 0. Parasite density upon enrollment in patients ranged between 2175–184,465 parasites/µl.

A total of 1500 samples from 196 subjects were included in this analysis, including 174 cases of adequate clinical and parasitological response and 22 microsatellite-confirmed recrudescences [11, 12]. Artemisinins remain efficacious in Angola and 97% of the analyzed participants had cleared microscopically-detectable parasitemia by Day 2, increasing to > 99% by Day 3.

### Laboratory analysis

A single 6 mm dried blood spot (DBS) punch (corresponding to 10 µl whole blood) was taken from each timepoint for a person’s series. Whole blood was eluted into buffer and analyzed for LDH, Aldo, and HRP2 concentration using previously-described methods [13, 14]. In brief, the 6 mm punch was placed into 200 µl blocking buffer overnight to allow elution [Buffer B: 0.5% polyvinyl alcohol (Sigma-Aldrich, St. Louis, MO, USA; P8136) 0.5% polyvinylpyrrolidine (Sigma-Aldrich; PVP360), 0.1% casein (Thermo Fisher Scientific, Waltham, MA, USA; 37528), 0.5% BSA (Sigma-Aldrich; A9418), 0.3% Tween-20, 0.05% sodium azide, and 0.01% *E. coli* extract to prevent non-specific binding]. For antigen detection, three unique bead regions (Bio-Plex COOH bead, BioRad, Hercules, CA, USA; 171506XXX) were individually coated by the EDC/Sulfo-NHS intermediate reaction with separate antibodies specific for each antigen to be captured: *Plasmodium* Aldo (12.5 µl/12.5 × 10^6^ beads, rabbit IgG anti-Aldo, Abcam, Cambridge, UK; ab207494), *Plasmodium* LDH (12.5 µl/12.5 × 10^6^ beads, mouse IgG anti-LDH, BBI Solutions, Cardiff, UK; BM355-Z8F7), and *P. falciparum* PfHRP2 (20 µl/12.5 × 10^6^ beads, mouse IgG anti-HRP2, Abcam; ab9206). For the multiplex antigen assay, the three unique bead regions were mixed together in 5 ml Buffer A (PBS, 0.5% BSA, 0.05% Tween20, 0.02% NaN3) so that 1500 of each bead region would be added per well in the assay plate. The beads were washed twice with 100 µL wash buffer (PBS, 0.05% Tween20) in the assay plate, and were incubated with 50 µl sample (corresponding to 2.5 µl whole blood) run in singlets in filter bottom plates (Millipore, Burlington, MA, USA; MABVN1250). Following a 90 min incubation with samples under gentle shaking at room temperature protected from light, plates were subsequently washed three times. Beads were incubated for 45 min with a 50 µl mix of detection antibodies: anti-pAldo (1:2000×, rabbit anti-Aldo, Abcam; ab207494), anti-pLDH [1:500× of 2:1:1 mixture (BBI Solutions BM355-P4A2:BioRad Pv-pLDH HCA156:BioRad Pf-pLDH HCA158), and anti-HRP2 (1:500×, mouse IgG anti-HRP2, Abcam, ab9203)]. All detection antibodies were previously biotinylated by Thermo Fisher Scientific EZ-Link Micro Sulfo-NHS-Biotinylation Kit according to the manufacturer’s protocol. Plates were again washed three times, and after a final 30 min wash step with reagent diluent, beads were washed once and resuspended in 100 µl PBS. After one minute on the shaker, plates were read on a Bio-Plex 200 instrument (BioRad, Hercules, CA, USA) by generating the median fluorescence intensity (MFI) signal for 50 beads in each unique region and then the mean fluorescence intensity of the MFIs among duplicates. The final measure, denoted as MFI-background (bg), was reported by subtracting a background signal defined as MFI values from beads on each plate only exposed to sample diluent during the sample incubation step.

To translate between a MFI-bg value and antigen concentration, standard curves for this relationship were calculated using recombinant antigens [14]. Recombinant LDH and HRP2 antigens were provided by Microcoat Biotechnologie GmbH (Bernried, Germany), and lyophilized preparations were rehydrated according to the manufacturer’s instructions. The *Plasmodium vivax-*specific isoform of Aldo was produced at the Centers for Disease Control and Prevention (CDC). Concentrations above 26,621 pg/ml for LDH, 464 pg/ml for Aldo, and 84 pg/ml for HRP2 were reported as positive.

### Statistical analysis

The log-transformed concentrations of each antigen were plotted over time. For each antigen, Kaplan–Meier curves were fit to estimate the time to clearance to below the level of detection (LOD) of the bead-based assay, stratifying by treatment outcome. Differences in antigen persistence by treatment outcome were compared using the log-rank test.

A Bayesian Markov Chain Monte Carlo (MCMC) algorithm was used to impute antigen concentrations for time points that were below the LOD of the bead-based assay for LDH (1111/1185 data points below the LOD), Aldo (723/1208), and HRP2 (7/945). In brief, the concentration at each time was modelled to follow a log-normal distribution, and a first-order kinetics model was fit to each individual’s antigen concentration time series data. After visual inspection showed an evident difference in clearance dynamics pre- and post-Day 2, separate pre-Day 2 ($\lambda_{1}$) and post-Day 2 ($\lambda_{2}$) clearance constants were estimated. The clearance constants, the standard deviation ($\sigma$), and the missing observations were sampled using an MCMC algorithm using Gibbs sampling. Uniform priors were used for $\lambda_{1}$, $\lambda_{2}$, and σ. The median and 95% credible intervals (CIs) were estimated from 9000 samples from the MCMC output, after discarding the first 1000 ‛burn-in’ samples. Data from patients with undetectable antigen at day 0 (*n* = 2/196 for LDH; *n* = 1/196 for Aldo; and n = 0/196 for HRP2) or time courses that were not monotonically declining (*n* = 24/196 for LDH; *n* = 43/196 for Aldo; and *n* = 73/196 for HRP2), defined as a > 25% relative or > 1000 pg/ml absolute increase in adjacent time points, were excluded from the estimation of clearance rates.

Statistical analysis was carried out in R version 3.3.2 (R Foundation for Statistical Computing, Vienna, Austria).

## Results

Pre-treatment Day 0 concentrations of all antigens were strongly correlated with initial microscopy-determined parasite densities, with the relationship strongest for LDH (*R*^2^ = 0.316), followed by Aldo (*R*^2^ = 0.245) and HRP2 (*R*^2^ = 0.168) (Additional file 1: Figure S1). Day 0 concentrations of Aldo (median: 35,758 pg/ml; geometric mean: 30,374 pg/ml) were orders of magnitude lower than concentrations of LDH (median: 555,255 pg/ml; geometric mean: 681,374 pg/ml) or HRP2 (median: 699,256 pg/ml; geometric mean: 787,603 pg/ml) (Table 1). On Day 0, the median ratio of HRP2 to LDH concentration was 1.2 (IQR: 0.6–2.0), of HRP2 to Aldo was 26 (8–84), and of LDH to Aldo was 21 (12–34).

**Table 1:** Clearance parameters for post-treatment dynamics of LDH, Aldo, and HRP2 proteins from *P. falciparum*-infected children in Angola

	LDH	Aldo	HRP2
Day 0 concentration (pg/ml), median (IQR)	555,255 (219,090–1,803,141)	35,758 (12,378–73,830)	699,256 (287,765–1,429,700)
Day 2 concentration (pg/ml), median (IQR)^a^	2559 (472–15,766)	1168 (594–2975)	421,035 (165,200–874,968)
Reduction from Day 0 to Day 2 (%), median (95% CI)^a^	99.7 (99.5–99.8)	96.32 (95.81–96.82)	54.63 (44.65–62.61)
Decay rate pre-Day 2 (λ_1_), d^−1^, median (95% CI)^a^	2.8 (2.6–3.0)	1.7 (1.6–1.7)	0.4 (0.30–0.49)
Decay rate post-Day 2 (λ_2_), d^−1^, median (95% CI)^a^	0.38 (0.31–0.45)	0.21 (0.20–0.23)	0.15 (0.14–0.15)
Variation in concentration (σ), median (95% CI)^a^	1.83 (1.83–1.83)	1.02 (0.94–1.13)	1.3 (1.2–1.3)
Half-life post-Day 2 (d), median (95% CI)^a^	1.8 (1.5–2.3)	3.2 (3.0–3.4)	4.8 (4.7–4.9)

*Abbreviations*: IQR: interquartile range; CI: credible interval; LDH: lactate dehydrogenase; Aldo: Aldolase; HRP2: histidine-rich protein 2; d: day

^a^Estimates calculated using observed and imputed values

**Fig. 1:**
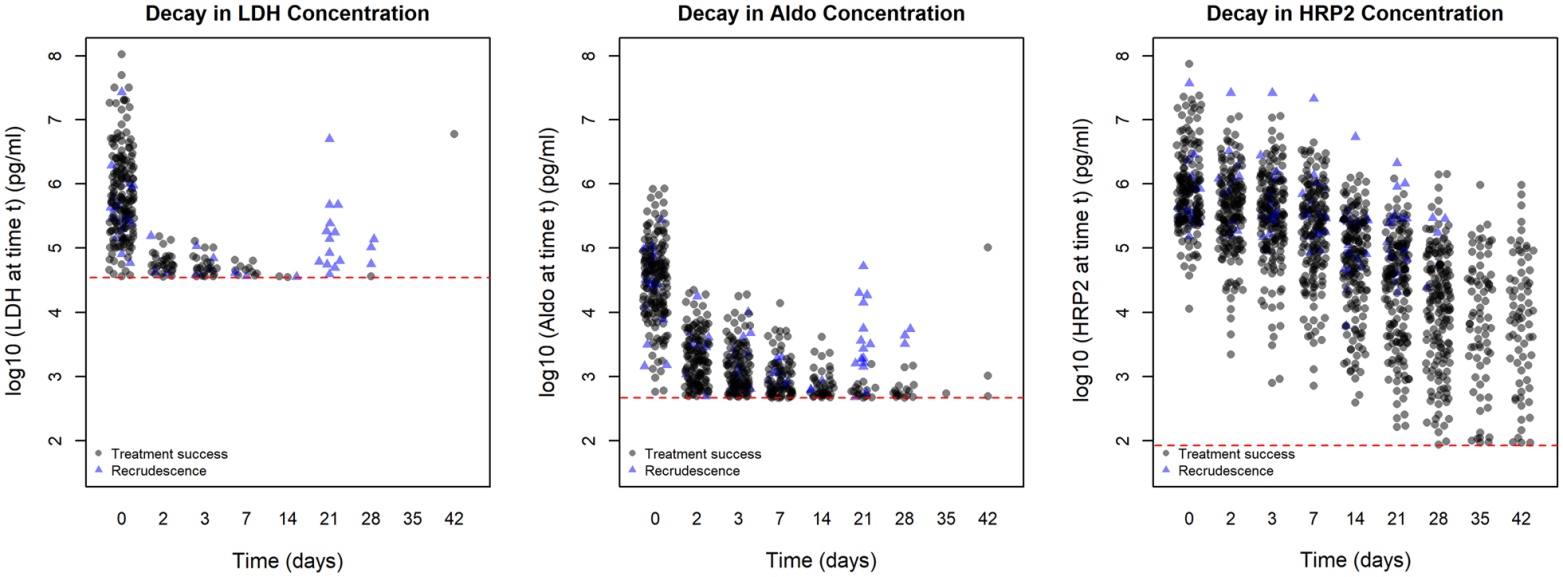
Decline in absolute malaria antigen following antimalarial treatment in Angolan children with uncomplicated *P. falciparum* infection. Dashed red line represents the limit of detection for each antigen

**Fig. 2:**
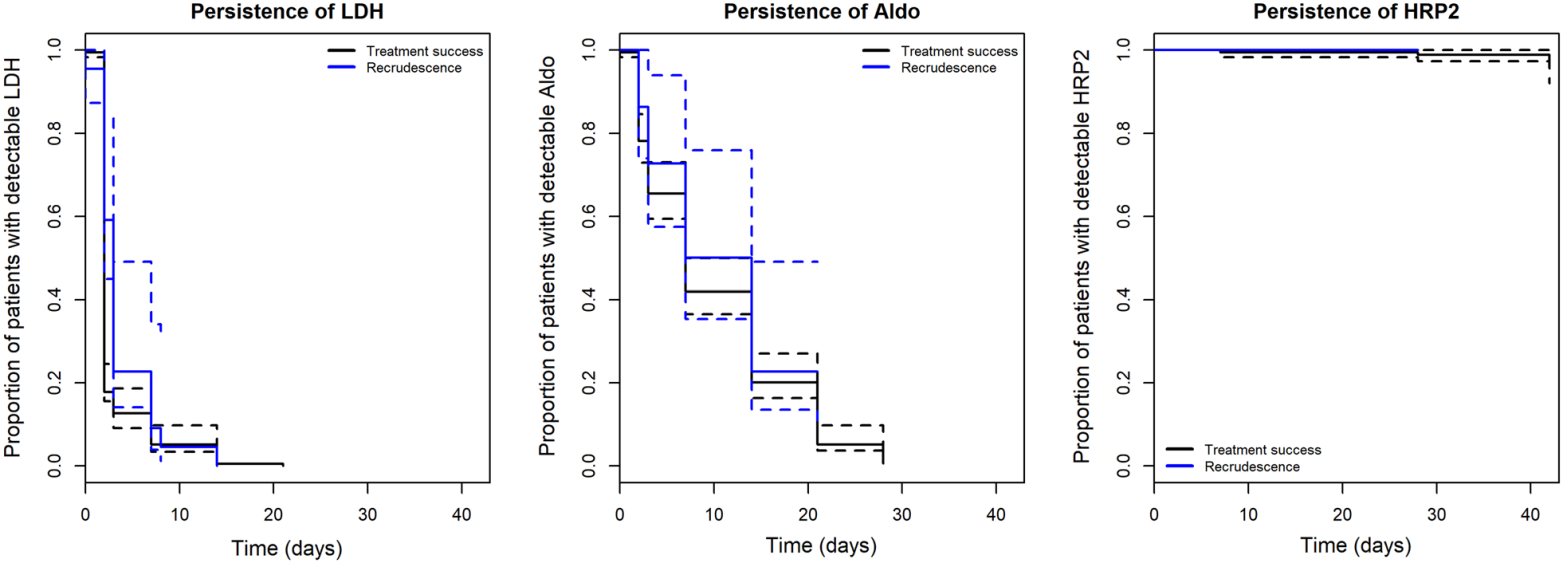
Persistence of malaria antigens following antimalarial treatment in Angolan children with uncomplicated *P. falciparum* infection. Dashed lines represent confidence intervals of the survival function

While the average decline between Day 0 and Day 2 HRP2 concentration was only 54.6%, it was 99.7% for LDH and 96.3% for Aldo (Fig. 1, Table 1). Nearly all patients had detectable HRP2 over the entire duration of follow-up, but levels of LDH and Aldo positivity declined over time (Fig. 2, Additional file 1: Figure S2). For both LDH and Aldo, rates of antigen persistence were higher in individuals that eventually recrudesced compared to those who did not, although this did not reach statistical significance (log-rank test *P*-value 0.2 for both LDH and Aldo).

Based on first-order kinetics models fit to post-Day 2 antigen concentrations, long-term clearance of LDH (half-life: 1.8 days, 95% CI: 1.5–2.3) was quickest, followed by Aldo (3.2 days, 95% CI: 3.0–3.4) and then HRP2 (4.8 days, 95% CI: 4.7–4.9) (Table 1). When compared to clearance after Day 2, clearance before Day 2 was: 7.4 times quicker for LDH, 8.1 times quicker for Aldo, and 2.7 times quicker for HRP2.

## Discussion

Fundamental differences in the clearance rates of LDH, Aldo, and HRP2 following microscopic clearance of *P. falciparum* infection were observed. On one extreme, nearly all LDH antigen (> 99%) is cleared within the first two days following antimalarial treatment, with the remaining amount being cleared at a much slower rate, with a half-life of 1.8 days. On the other extreme, more than half of the Day 0 HRP2 antigen is still present at Day 2 and HRP2 is cleared at an even slower rate after that (half-life of 4.8 days). These post-Day 2 clearance estimates for the HRP2 antigen are quite similar to previous estimates [3], confirming the long-term persistence of this antigen in the human host. Aldo takes an intermediate position in the spectrum between the other two antigens, and these differences hint at inherent biological differences in the clearance of these exogenous proteins from humans. LDH and Aldo are both expressed inside the parasite and are crucial for the functioning of the parasite cell [15, 16]. In contrast, HRP2 is largely secreted outside the parasite [17], and its biological function is not known. Its persistence is largely due to accumulated HRP2 in once-infected red blood cells [18]. In this context, it is not surprising that the vast majority of LDH and Aldo is cleared concurrent with clearance of the parasites themselves, and that the unbound extracellular HRP2 does not show such a dramatic decline in the first two days. Moreover, the ratios of pre- and post-day 2 clearance rates for LDH and Aldo are very similar, suggesting the two antigens might share a similar mechanism of clearance. The link between LDH and Aldo concentration and the presence of the parasite itself is consistent with the previous observation that LDH and Aldo levels are more closely associated with current parasite density, compared to HRP2, which can be interpreted more accurately as a measure of cumulative parasite load over the course of the infection [4, 19, 20].

A further nuance is introduced by apparent differences in antigen expression. The pre-treatment concentration of Aldo was 20–30 times lower than LDH and HRP2, which confirms previous results showing lower expression of Aldo compared to LDH and HRP2 [1]. These lower absolute levels of the protein during blood stage infection should be considered in the design of tests using Aldo as a diagnostic target.

Ultimately, antigen positivity in individuals living in endemic areas should be interpreted in the context of the differences in post-treatment clearance. Previous analysis of outpatients presenting to Angola health facilities showed that all patients with detectable levels of all three antigens were also positive for parasite nucleic acids, whereas PCR positivity rates were lower in individuals just positive for Aldo and HRP2 and much lower for HRP2 positivity alone [14]. Moreover, previous analytical work has shown that while presence of HRP2 is not necessarily synonymous with acute febrile disease, presence of LDH and Aldo at levels detectable through the bead-based assay used here are predictive of parasite densities high enough to provoke clinical disease [20].

These results are only from one country and are not necessarily generalizable. Rapid clearance of LDH and Aldo, combined with a higher assay LOD for these antigens, limit the ability of a definitive statement regarding the clearance dynamics of these antigens to be drawn from this study. In particular, it was not possible to test whether a second-order kinetics model provided a better fit than a first-order kinetics model. However, in relation to the slow clearance dynamics of the HRP2 antigen, these two pan*-Plasmodium* targets would clearly be more indicative of an active infection since they were absent in almost all patients within 7 days of starting an ACT regimen. Finally, the observation of a substantial number of patients showing post-day 7 spikes in antigen concentration could be due to false positive readings on the bead-based assay, or evidence of undetected new infections or recrudescences during the period of follow-up, and warrants further investigation.

## Conclusions

Differential clearance of malaria antigens used as targets for diagnostic testing raises the possibility of inferring past history of the disease course in an individual based on the presence and quantity of the different antigens. HIV programs have for years capitalized on the differences in the dynamics of how certain antibodies appear in the host to distinguish recent infections from older infections, allowing estimation of incidence from cross-sectional survey data [21]. Analogously, specimens from household community surveys in malaria-endemic countries could be potentially analyzed by the multi-antigen bead-based assay to distinguish past infections from active ones, and possibly make inferences regarding treatment rates preceding the survey. Findings from this study should be confirmed in other settings and countries.

## Additional file

**Additional file 1: Figure S1.** Relationship between pre-treatment (Day 0) microscopically-determined parasite density and LDH, Aldo, and HRP2 concentrations in Angolan children with uncomplicated *P. falciparum* infection. **Figure S2**. Decline in absolute malaria antigen following antimalarial treatment in Angolan children with uncomplicated *P. falciparum* infection. Dashed red line represents the limit of detection for each antigen. Each line represents a time course for a single participant.

## Data Availability

All data are available from the authors upon request.

## Abbreviations

AL: artemether–lumefantrine
Aldo: aldolase
ASAQ: artesunate–amodiaquine
CDC: Centers for Disease Control and Prevention
DP: dihydroartemisinin–piperaquine
HRP2: histidine-rich protein 2
LDH: lactate dehydrogenase
LOD: level of detection
MCMC: Markov chain Monte Carlo
MFI-bg: median fluorescence intensity-background
RDT: rapid diagnostic test
