# Supplementary material for: Clearance dynamics of lactate dehydrogenase and aldolase following antimalarial treatment for Plasmodium falciparum infection
Source: Parasit Vectors. 2019 Jun 10;12:293. doi: 10.1186/s13071-019-3549-x (PMC6558726; doi:10.1186/s13071-019-3549-x)
Supplement: Supplementary file 1 — Additional file 1: Figure S1. Relationship between pre-treatment (Day 0) microscopically-determined parasite density and LDH, Aldo, and HRP2 concentrations in Angolan children with uncomplicated P. falciparum infection. Figure S2. Decline in absolute malaria antigen following antimalarial treatment in Angolan children with uncomplicated P. falciparum infection. Dashed red line represents the limit of detection for each antigen. Each line represents a time course for a single participant. [file 13071_2019_3549_MOESM1_ESM.pdf]

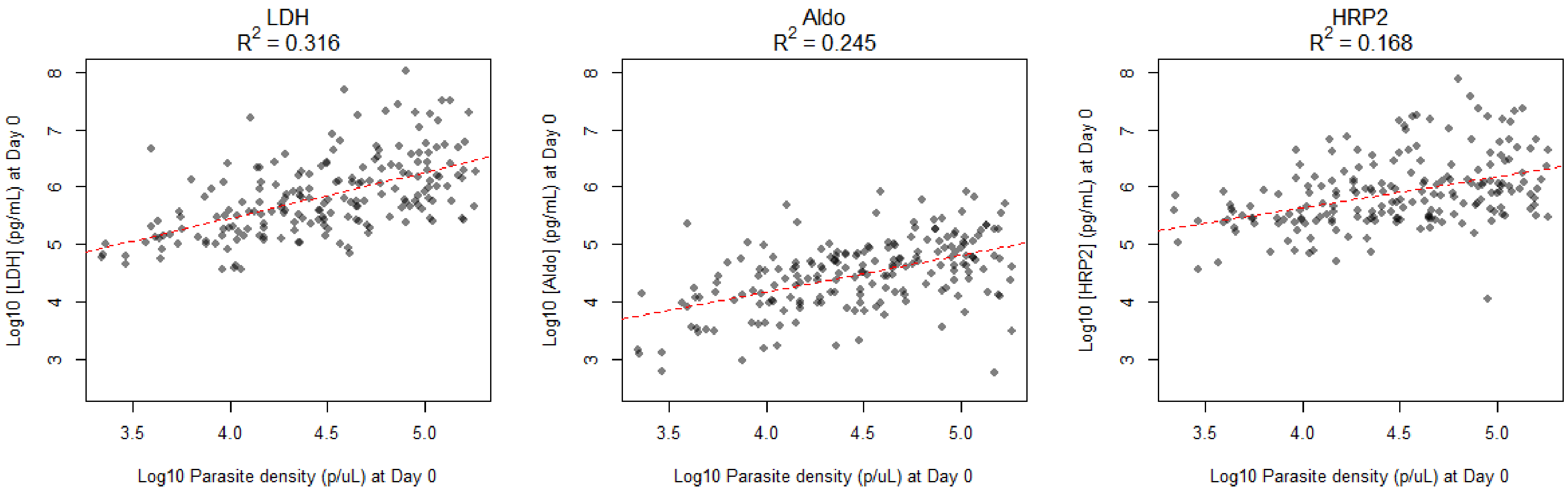

Figure S1. Relationship between pre-treatment (Day 0) microscopically-determined parasite density and LDH, Aldo, and HRP2 concentrations in Angolan children with uncomplicated *P. falciparum* infection.

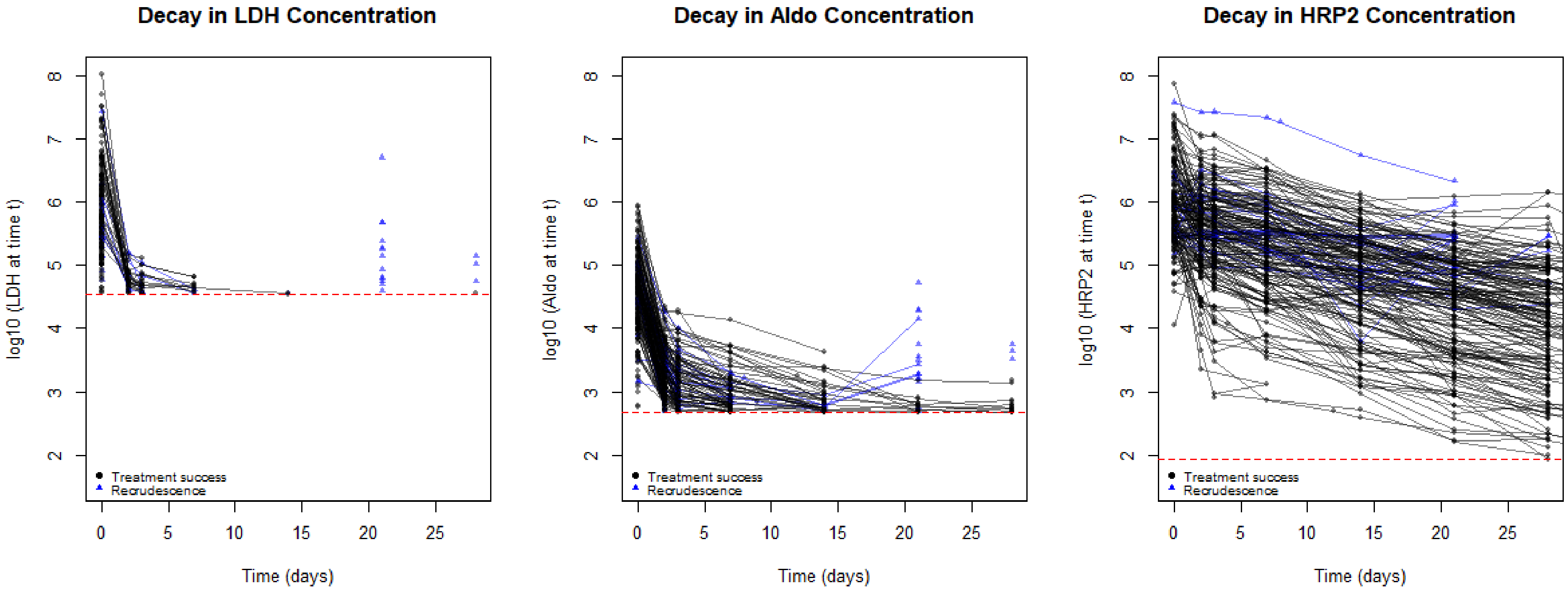

Figure S2. Decline in absolute malaria antigen following antimalarial treatment in Angolan children with uncomplicated *P. falciparum* infection. Dashed red line represents the limit of detection for each antigen. Each line represents a time course for a single participant.
